# Abnormal Neurologic and Motor Function in Newborns Treated for Congenital Syphilis

**DOI:** 10.3390/idr17020034

**Published:** 2025-04-16

**Authors:** Bruna Silva, Luciana Friedrich, Graziela Biazus, Renata Bueno, Carla Almeida

**Affiliations:** 1 Physical Education, Physioterapy and Dance School, Federal University of Rio Grande do Sul, Porto Alegre 90010-150, Brazil; brunavitoriar99@gmail.com (B.S.); renatabueno97@gmail.com (R.B.); carlaskilhan@gmail.com (C.A.); 2 Pediatrics Department, Federal University of Rio Grande do Sul, Porto Alegre 90010-150, Brazil; 3 Neonatology Service, Hospital de Clínicas of Porto Alegre, Porto Alegre 90035-903, Brazil; 4 Physioterapy Service, Hospital de Clínicas of Porto Alegre, Porto Alegre 90035-903, Brazil; gbiazus@hcpa.edu.br

**Keywords:** congenital syphilis, newborns, neurological examination

## Abstract

**Background:** Congenital syphilis (CS) is a transplacental infection that can lead to many long-term sequelae when not adequately treated; however, knowledge about the motor and neurological signs that newborns (NBs) with CS may present is scarce. **Objective:** The aim of this study was to describe the results of neurological assessment scales and general movements in NBs with CS in the first days of life. **Methods:** In this case-series, the Hammersmith Neonatal Neurological Examination (HNNE) and General Movements Assessment (GMA) scales were used to evaluate NBs under treatment for CS in a public Brazilian hospital in the first days of life. **Results:** The sample consisted of 11 NBs, with a mean birth weight of 3140.5 g, and an Apgar score at 5 min of 8.3. Among the 11 mothers, 4 (36.4%) had fewer than six prenatal visits and 5 (45.5%) did not receive any syphilis treatment. All NB (100%) were asymptomatic, with normal long bone X-rays and cerebrospinal fluid analysis. The mean score on the HNNE was 22 (suboptimal/abnormal). Eight NBs (72.7%) showed abnormalities on GMA scale (with six [54.5%] being mildly abnormal and two [18%] definitely abnormal). Only three NBs (27.3%) returned for outpatient follow-up. **Conclusions:** Neurological and motor evaluations were abnormal in most of the asymptomatic NBs under treatment for CS in the first days of life, when assessed through specific scales (HNNE and GMA). Most mothers did not receive adequate treatment for syphilis during pregnancy, and there were important deficiencies in post-discharge follow-up. Further studies are needed to confirm these findings and investigate whether the observed abnormalities are linked to biological or environmental factors during pregnancy.

## 1. Introduction

Congenital syphilis (CS) is part of the ZSTORCH group of infections (syphilis, toxoplasmosis, rubella, human immunodeficiency virus, hepatitis B and C, cytomegalovirus, herpes virus, and zika virus), most of which are associated with an increased risk of neonatal morbidity and mortality, as well as neurological sequelae [1,2]. The incidence of CS in Brazil was 9.9/1.000 live births in 2022 [3]. When untreated at birth or within the first 3 months of life, CS can lead to systemic, cutaneous, hematological, musculoskeletal, kidney, and respiratory manifestations and sequelae, neurological impairment, and developmental delay [4]. However, the literature is scarce regarding motor and developmental delays in this population, especially at a young age. The early detection of potential problems allows for the early beginning of medical and physiotherapeutic intervention, using the neuroplasticity window and promoting better recovery [5].

Brazilian Ministry of Health (MoH) guidelines provide a specific organogram to manage the diagnosis and treatment for both mother and neonate [4]. During pregnancy, a treponemal rapid test should be taken in all pregnancies in the first trimester. Treponemal rapid tests can be performed outside a laboratory setting, with minimal training and no equipment, using a small amount of whole blood collected via finger prick. The test has high sensitivity (85–97.7%) and specificity (92.8–98%). These results are comparable to the best laboratory-based tests. When negative, it should be repeated at least in each trimester of pregnancy. When positive, a nontreponemal test (usually Venereal Disease Research Laboratory—VDRL) should be immediately taken in order to know pre-treatment titers and to follow-up the titers after proper treatment. Maternal syphilis treatment during pregnancy is considered adequate as follows:

- Mandatory use of Benzathine Penicillin (any other drug is not allowed during pregnancy due to the absence of a capacity for crossing the placenta).
- Dosing: 2,400,000 UI (2 ampoules), intramuscularly, for 3 consecutive weeks (7-day interval), with a total dosing of 72,000 UI.
- Treatment should be initiated up to 30 days before delivery.
- Monthly VDRL tests should be taken, and there should be a decrease in seric maternal VDRL titers by at least two dilutions within three months, or four dilutions within six months after the end of treatment [4].

The diagnosis of CS in the newborn is also made accordingly to the Brazilian MoH protocol, based on the adequacy of the mother treatment (complete or not), seric VDRL titers, the presence of abnormalities in physical examination (systemic, neurological, cutaneous, hematological, pulmonary, renal, and musculoskeletal, among others), abnormalities in long bone X-rays, and the analysis of cerebrospinal fluid (to assess for neurosyphilis). If CS is diagnosed or highly suspected, treatment is conducted through the administration of a 10-day course of endovenous Crystalline Penicillin on a hospital basis [4].

Studies have shown that between 46 and 60% of sexual partners of mothers infected with syphilis (mainly in primary and secondary stages) are probably also infected [6]. Therefore, even when partners have a negative rapid test, they may be in the “immunological window”, if recently exposed (less than 90 days). In this situation, it is advisable to apply a single-dose Benzathine Penicillin. No or incomplete treatment of a partner may increase by 50% the risk of fetal infection.

There is no described Penicillin-resistant Treponema pallidum. Some mutations are described that make the spirochete resistant to macrolides and tetracyclines, with Penicillin being the only choice of treatment during pregnancy, even because it is the only drug that can cross the placenta to prevent fetal infection. Any other antibiotics prescribed during pregnancy would be labeled as “non-adequate treatment” for mothers [7,8,9].

The surveillance of early motor development can facilitate the detection of motor delays or disturbances (i.e., in postural and/or movement control, abnormal movement patterns, and muscle tone), leading to early interventions aimed at preventing future structural or functional disorders from developing. According to recent recommendations from the American Academy of Pediatrics, health professionals should routinely monitor early development and conduct standardized developmental screenings in the first year of life [10]. Simple, low-cost tests with high sensitivity can indicate the motor and neurological profiles of newborns (NBs). Screening scales are ideal for patients with CS.

The Hammersmith Neonatal Neurological Examination (HNNE) is a standardized assessment instrument used to verify the neurologic function and integrity of the central nervous system (CNS) from birth until 2 months of corrected age, identifying early signs of cerebral palsy. It is a simple, quantifiable, neurologic examination with reliability between examiners being 96%. It can be completed in 10–15 min and it allows for the early identification of neurological development delays [11]. It is a prognostic evaluation, counted by added points. When the baby does not reach the required points, the classification is termed “suboptimal” or “abnormal”.

The General Movement Assessment (GMA) is an instrument to observe spontaneous movements from birth until 5 months of corrected age. It identifies neurological abnormalities and is considered a diagnostic test, not needing a specific validation in Brazil. Newborns are classified into “typical” or “pathologic” movements [12].

Both scales are compatible with the reality of public health in Brazil, where simple, low-cost tests are needed to identify the motor and neurologic profiles of NBs with CS [13].

Studies by Padilha et al. [14] and de Freitas et al. [6] observed abnormal results of both scales in a sample of NBs admitted to an intermediate neonatal care unit (NU) in a specialized hospital for maternal and child care. They found that CS was the second leading cause of hospitalization, with prematurity being the first. Patients being treated for CS had most of their results classified as “pre-pathological/pathological” in the GM evaluation and had “suboptimal” results in HNNE. These studies have motivated the development of the current research, with a sample of NBs being treated for CS.

The aim of this study was to describe the results of neurological (HNNE) and motor scales (GMA) in NBs with CS hospitalized in the NU of a tertiary hospital in Brazil.

## 2. Materials and Methods

This study is a case-series in NBs being treated for CS. The sample consisted of NBs with CS hospitalized in the NU of a tertiary reference public hospital in Porto Alegre, a state capital in southern Brazil. This city has the highest rates of syphilis detection in pregnant women and CS in the country, which are up to three times higher than the national average [3]. The sample was recruited sequentially, including all live births with CS admitted for treatment between August and December 2023.

The research excluded NBs with other coexisting congenital ZSTORCH infections that could lead to early neurologic problems (congenital zika virus, toxoplasmosis, rubella, cytomegalovirus, and herpes virus infections), major congenital anomalies, or genetic syndromes, which could present motor or neurological delays as their main characteristics. Additionally, preterm NBs less than 34 weeks were excluded due to the increased risk of clinical complications and neurological deficits [15].

The instruments used were as follows: (1) HNNE, a neurological assessment used as a diagnostic method for cerebral palsy [11]. The scoring on this scale is based on the gestational age of the NB and the classification is determined by the sum of points. Scores equal to or above 30.5 for full-term NBs and above 26 for preterm NBs are considered “optimal”. Scores below these thresholds are classified as “suboptimal” or “abnormal” [16]. (2) GMA, which evaluates the central nervous system (CNS) through observation of the NB’s spontaneous movements [17]. These GMs change according to the NB’s maturation, thus varying based on gestational age [12]. The classification of GMs varies between “normal optimal”, “normal suboptimal”, “mildly abnormal”, and “definitely abnormal”, based on the complexity, variability, and fluency of the NB’s spontaneous movements observed for 1 (one) minute.

The ABEP (Brazilian Association of Research Companies) classification was also used. This is a questionnaire for assessing Brazil’s economic classification, which ranges from ‘A’ to ‘E’, where ‘A’ represents a higher socioeconomic condition, down to ‘E’, which corresponds to the lowest socioeconomic condition [18].

According to the Brazilian Ministry of Health (MoH) protocol [4], gestational syphilis treatment was considered adequate as follows:

- Mandatory use of Benzathine Penicillin (any other drug is not allowed during pregnancy due to the absence of a capacity for crossing the placenta).
- Dosing: 2,400,000 UI (2 ampoules), intramuscularly, for 3 consecutive weeks (7-day interval), with a total dosing of 72,000 UI.
- Treatment should be initiated up to 30 days before delivery.
- Monthly VDRL tests should be taken, and there should be a decrease in seric maternal nontreponemal titers by at least two dilutions within three months, or four dilutions within six months after the end of treatment [4]. The nontreponemal test used for this purpose in our setting is the VDRL (Venereal Disease Research Laboratory) test.

The diagnosis of CS in the newborn was also made accordingly to the Brazilian MoH protocol, based on the treatment of the mother (complete or not), seric nontreponemal titers (VDRL), the presence of abnormalities in physical examination (systemic, neurological, cutaneous, hematological, pulmonary, renal, and musculoskeletal, among others), abnormalities in long bone X-rays, and the analysis of cerebrospinal fluid (to assess for neurosyphilis). Upon diagnosis of CS, the NB was referred to the NU for treatment with Crystalline Penicillin for 10 days.

The mother’s and NB’s medical records were analyzed to collect demographic, gestational, maternal, and neonatal data. The ABEP questionnaire was administered to the NB’s caregiver to assess the family’s socioeconomic status and the educational level of the household head. Evaluations using the HNNE and GMA scales were then conducted during the NB’s hospitalization.

Regarding the HNNE scale, a score equal to or above 30.5 for full-term NBs or 26 for preterm NBs was considered “optimal”. Scores below these values were classified as “suboptimal” or “abnormal”. For the GMA scale, results were categorized as follows: “normal optimal” (corresponding to “three plus” in complexity and variability and “one plus” in fluency), “normal suboptimal” (“two plus” in complexity and variability and “one minus” in fluency), “mildly abnormal” (“one plus” in complexity and variability and “one minus” in fluency”), and “definitely abnormal” (“one minus” in all three criteria). We considered “mildly abnormal” and “definitely abnormal” as abnormal classifications. Extreme alerts must be carried out when “cramped synchronized movements” (CSMs) are observed. These movements are defined as stiffness and a lack of fluency and gracefulness in movements, including the simultaneous contraction and relaxation of muscles, with a high predictive value for cerebral palsy.

Data were analyzed using the Statistical Package for the Social Sciences (SPSS 20.0 IBM, Boston, USA, 2009). Quantitative variables were described using means and standard deviations, and qualitative variables were described using frequencies and percentages. “*p*” values were considered statistically significant if <0.05.

Descriptive analytics were taken in SPSS 20.0 software, regarding qualitative and quantitative data. Nominal data were analyzed through relative and absolute frequencies. Continuous data were analyzed through mean and standard deviation.

The research project was approved by the Institutional Research Ethics Committee. All parents and/or legal guardians of the participants signed an informed consent form.

## 3. Results

### 3.1. Sample

The sample consisted of 11 NBs who were admitted to the NU for the treatment of CS during the study period. There was a significant decrease in admissions from September to October due to technical/administrative reasons in the NU, which consequently led to a much smaller number of admissions for all reasons, including CS, compared to usual periods.

Maternal and neonatal demographic data can be observed in Table 1.

### 3.2. Demographic, Maternal, and Neonatal Characteristics

All NBs were born full-term and with a high Apgar score. Only one NB (9%) was classified as small for gestational age (SGA); the others were appropriate for gestational age (AGA). Regarding prenatal care appointments, four mothers (36.4%) did not attend the minimum recommended six visits according to the Ministry of Health guidelines, with two of them (18.2% of the total) having no prenatal visits at all.

Regarding maternal treatment, 10 women (90.9%) reported receiving a syphilis diagnosis during this pregnancy. Among them, five (45.5%) did not undergo any treatment for the disease during the current pregnancy; two (18.2%) received incomplete treatment; and four (36.3%) completed all doses but did not achieve a decrease in VDRL titers, leading to the diagnosis of CS in their NB. Only five sexual partners (45.5%) also received treatment.

Three pregnant women (27.3%) used psychoactive substances/illicit drugs during pregnancy, and two (18.2%) were coinfected with HIV.

All NBs included in the study were asymptomatic and had normal blood counts, normal long bone radiographs, and normal cerebrospinal fluid analysis, indicating the absence of neurosyphilis. No infants were excluded during the period due to prematurity, genetic syndromes, or major malformations.

Regarding socioeconomic evaluation, six families (54.5%) fit into category C2 (lower middle class); the remaining categories can be observed in Table 2.

### 3.3. Neurologic Assessment Scale (HNNE)

All 11 NBs (100%) showed abnormal neurological assessment using the HNNE scale. The mean score was 22, considered “suboptimal/abnormal”. Table 3 shows the scale scores for the evaluated NBs. Table 4 demonstrates the most frequently abnormal items in the scale application (out of a total of 34 items).

### 3.4. General Movements Scale

Regarding the GMA scale, eight NBs (72.7%) showed abnormalities, with six (54.5%) classified as “mildly abnormal” and two (18.2%) as “definitely abnormal”. Among the six cases classified as “mildly abnormal”, three of these mothers (50%) used psychoactive substances or illicit drugs during pregnancy (marijuana, cocaine, crack, or alcohol), and one (16.7%) was seropositive for HIV.

Three out of the eleven NBs (27.3%) were referred for institutionalization through judicial proceedings due to the termination of parental rights, being discharged from the hospital directly to the shelter.

### 3.5. Post-Discharge Follow-Up

After hospital discharge, among the 11 evaluated patients, only 3 (27.3%) returned for outpatient follow-up at the routine CS outpatient clinic after 3 months. These three patients were the NBs who were institutionalized after hospitalization; therefore, they were brought to the appointment by the shelter caregivers.

Among the two NB who had the lowest scores in the assessment of GM, classified as “definitely abnormal”, one did not return for follow-up at the outpatient clinic, and the other was referred to an institution due to a mother who was homeless, a user of psychoactive substances, and seropositive for HIV without treatment. This NB was the only SGA in the study. When he attended the clinic, the caregivers were forwarded to undergo physiotherapy for the developmental issues.

Among the six neonates with “mildly abnormal” GMA scores, only two attended the outpatient clinic for follow-up, both of whom came from institutions. One of them was already in an adoptive family.

## 4. Discussion

The majority of the NBs in this study presented abnormal results in the GMA and HNNE neurologic and motor scales, assessed in the first days of life. These results were observed despite all neonates being full-term, having good birth weights, high Apgar scores, asymptomatic CS, and no neurosyphilis. Similar findings were observed in other studies using similar patients (all NBs admitted to an intermediate neonatal care unit), showing that patients being treated for CS had worse neurologic results than preterm ones [6,14]. These studies applied separately to both scales in NBs admitted in a conventional neonatal care unit in tertiary hospitals in the same city of the current study. The authors described that despite prematurity being the main leading cause of hospitalization, newborns being treated for CS presented with abnormal results in both scales, and many of these findings were also observed with 52 corrected weeks of age, with lower scores even when compared to preterm NBs.

Both scales used are sensitive and validated for the target population. Regarding the scope of this study, it should be mandatory that infants that belong to this population be neurologically assessed during the first days of age and followed prospectively during the first year of life.

According to Hadders-Algra [5], “neurodevelopment peaks in the second half of gestation and at three months of postnatal age, but remains high until the first year of life”. Therefore, the early detection of developmental disorders allows for early intervention, leading to more successful outcomes due to high neuroplasticity during this period.

There are few studies involving the simultaneous testing of both scales in NBs with CS. The HNNE scale is used to classify the risk of cerebral palsy, with an inter-rater reliability of 96%. All NBs in this study showed abnormalities in the scores of this scale. Chaves et al. also found “suboptimal/abnormal” scores in a small group of neonates with CS and observed, using the Hammersmith Infant Neurological Examination (HINE), that these abnormalities were maintained up to 52 weeks of age in these infants, suggesting that neurological changes may persist at least until the end of the first year of life.

The GMA scale evaluates the CNS through the observation of the NB’s spontaneous movements [17]. A study by Padilha et al. [14] evaluated the GM of NBs hospitalized in an NU and found that half of the ones with CS had abnormal results in these tests. In the present study, 72.7% of the NBs showed abnormalities, even mild ones, in the GMA, with two of them (18.2%) considered as “definitively abnormal”. Additionally, one NB presented with CSMs, which are sudden block-like movements where the limbs move in synchronous rigidity. These movements indicate a loss of supraspinal control and are considered pathological, with a high association with cerebral palsy when they occur frequently, and may also be associated with abnormalities in cerebral magnetic resonance imaging [19].

In recent decades, there have been much higher rates of acquired and gestational syphilis in middle- and low-income countries than in high-income ones. Fragilities in adherence to treatment are associated with the lack of awareness of the disease consequences (in mother and child), as well as the trivialization of safe sexual practices, resulting in a high prevalence of transmission and re-infection, often associated with low levels of family education and drug addiction [20,21,22].

According to data from the Department of Chronic Conditions and Sexually Transmitted Infections of Syphilis in Brazilian Municipalities in 2022, inadequate treatment [3] during pregnancy was reported in 81% of NBs who needed treatment for CS. In the present study, although 63.6% of mothers had adequate prenatal care with six or more visits, as recommended by the Brazilian Ministry of Health, 45.5% did not receive any treatment for syphilis during the current pregnancy. These findings are consistent with a study by Rocha et al. [23], where despite prenatal care and testing for syphilis, only 7.9% of mothers were adequately treated during pregnancy. Furthermore, Araújo et al. [24] found VDRL titers at delivery greater than 1:8 in 79.8% of cases in mothers and 14.4% in newborns, which was associated with worse outcomes in the neonates [24].

Despite the high coverage of antenatal assessment in our country, there are still many barriers to an adequate access of gestational syphilis treatment in Brazil, highlighting our social inequities [25]. The fragility of the dynamics of health services and the low quality of antenatal assessments favor high prevalences of syphilis, hindering its diagnosis and treatment [26]. A national study showed that 79% of mothers who had their NBs treated for CS in the neonatal period had accessed antenatal appointments. Of those, 55% had their gestational syphilis diagnosed during the appointments and 66% did not have their sexual partners treated. Even with antenatal care and receiving their diagnosis during pregnancy, most of them did not receive adequate treatment [27].

These data suggest the importance of more effective monitoring of pregnant women with syphilis, through stricter prevention, treatment, and follow-up actions. It is known that the treatment and correct monitoring of these pregnant women, as well as the treatment of their sexual partners, are crucial in reducing pregnancy complications and the prevalence of syphilis in the fetus and NB.

It is important to emphasize that the correct treatment of syphilis in pregnant women involves several criteria. The woman needs to be well informed that the three doses of Penicillin must be administered with an interval of 7 to a maximum of 9 days. Untreated sexual partners can increase the risk of re-infection in the pregnant woman by up to 50%. Even with correct treatment, depending on the level of circulating treponemes, gestational age, and other comorbidities (e.g., HIV coinfection or maternal neurosyphilis), the therapeutic failure rate can reach up to 14%, significantly increasing the risk of syphilis in the fetus despite the mother receiving “adequate” treatment. This underscores the importance not only of treatment but also of closely monitoring these pregnant women for the neonatal outcome [4].

Another important issue addressed in this study, which aligns with the inadequate treatment of gestational syphilis, is the socioeconomic status of families, which directly influences access to healthcare services [28]. In this study, the vast majority (90.9%) of families were classified in socioeconomic classes C, D, and E, in line with several studies reporting socioeconomic vulnerability and low maternal education as recurring factors associated with cases of CS [28,29]. Regarding motor development, a study by Delgado et al. evaluated infants aged 4 to 17 months and found that socioeconomic factors, such as receiving government benefits and low maternal education, are independent risk factors for neurodevelopmental impairment.

The lack of adherence to post-discharge outpatient follow-up, whether due to access or mobility difficulties, or a lack of understanding of the prognosis and potential sequelae of CS, had a significant impact in this study. Among the 11 patients studied, the only 3 patients who attended the first follow-up appointment were those referred to and brought by judicial institutions. Also striking is the high prevalence of mothers with substance abuse disorders and coinfection with HIV, factors possibly related to poor adherence to prenatal care, the lack of treatment during pregnancy, and the inadequate follow-up of the NBs after discharge.

It is important to note that *Treponema pallidum* is still not resistant to Penicillin. All international guidelines advise Penicillin as the first line of treatment in all stages of the disease. There are some described mutations that make *Treponema pallidum* resistant to macrolides and tetracyclines, but there is no described Penicillin resistance in any study [7,8,9]. In addition, all international guidelines, including those of the CDC and the Brazilian Ministry of Health, advise only Penicillin for the treatment of gestational syphilis, since it is the only drug that crosses the placenta and can prevent fetal infection. In total, 14% failure of treatment during pregnancy is described, generally because of late treatment, maternal immunodeficiency (HIV), maternal neurosyphilis, or an already infected fetus [4,30].

This study has some limitations, including a small sample size and the absence of a control group not exposed to gestational syphilis, which prevented confirming that the findings were due to syphilis itself rather than other in utero environmental issues. There are no known studies evaluating the results of these scales in a “normal” population of NBs in our country. However, two studies of the same group of professionals have published the results of the scales in neonates admitted to an intermediate neonatal care unit (thus being at risk of impaired neurodevelopment), with findings of abnormal results in both scales in full-term NBs being treated for congenital syphilis, even more abnormal than in preterm infants [6,14].

The small number of participants limits interpretability and statistical analysis. In addition, it was not possible to perform association tests between abnormal results and other maternal or neonatal risk factors. It was also not possible to demonstrate the relationship between socioeconomic variables and abnormalities in scale results, despite previous studies showing such associations. Furthermore, our study was not blinded, as the investigators conducting and scoring the behavioral tests on the babies knew that the babies had congenital syphilis. Therefore, the investigators could be unconsciously biased in the assessments. The follow-up of a larger sample of patients, together with a control group, is being conducted at 3 and 6 months of age, to observe if these abnormalities persist during the first year of life.

## 5. Conclusions

Abnormal neurologic tests were found in NBs during CS treatment in the first days of life. Most of the families belonged to lower socioeconomic classes and there was a high prevalence of mothers who did not receive any treatment for syphilis. Additionally, the majority of families did not attend the post-discharge follow-up for their NBs. Further controlled studies with a larger number of patients are recommended to confirm these findings and determine whether the observed abnormalities are more closely associated with biological or socioeconomic factors, as well as if they persist through the first year of life.

There is an urgent need for preventive measures and better monitoring of pregnant women with syphilis, since the early referral of any NB with suspected neurodevelopmental delay is crucial. The identification of abnormalities before hospital discharge can prevent future expenses related to avoidable complications and capitalize on the period of heightened neuroplasticity.

## Figures and Tables

**Table 1:** Maternal and neonatal demographic data (*n* = 11).

	Mean ± SD	Min–Max
No. of prenatal appointments	7 ± 5.2	0–16
GA (weeks)	39.2 ± 1.3	37–41
Birth weight (g)	3140 ± 457	2230–3925
Apgar at 5th minute	9.1 ± 0.8	8–10

Legend: SD: standard deviation; GA: gestational age; g: grams; Min: minimum; Max: maximum.

**Table 2:** Socioeconomic evaluation (*n* = 11).

ABEP Categories	Frequency	Percentage (%)
B2 (upper middle class)	1	9.1
C1 (middle class)	2	18.2
C2 (lower middle class)	6	54.5
D/E (lower classes)	2	18.2

Legend: ABEP: Brazilian Association of Research Companies.

**Table 3:** Results of the HNNE scale (*n* = 11).

	Max Score of the Scale	Mean ± SD	Min-Max
Total HNNE score	34	22 ± 2.4	19–26
Posture and tone	10	4.3 ± 1.6	2–7
Tone patterns	5	4.5 ± 0.5	4–5
Reflexes	6	4.6 ± 0.8	4–6
Movements	3	1.4 ± 0.9	0–3
Abnormal signs	3	2.4 ± 0.5	2–3
Behavior	7	4.8 ± 1.3	3–7

Legend: SD: standard deviation; Min: minimum; Max: maximum.

**Table 4:** Most frequently abnormal items of HNNE scale (*n* = 11).

	**N (%) Abnormal**
Posture 1	11 (100)
Spontaneous movements 1 (quantitative)	8 (72.7)
Spontaneous movements 2 (qualitative)	9 (81.8)
Ventral suspension	9 (81.8)
Tremors	3 (27.3)
Cramped synchronized movements	1 (9.1)

## Data Availability

The authors declare that all data generated or analyzed during this study are included in this published article. Some personal patients’ data are not publicly available but will be available from the corresponding author on reasonable request.

## Abbreviations

CS	Congenital syphilis
HNNE	Hammersmith Neonatal Neurological Examination
GMA	General Movements Assessment
NB	Newborn
GM	General movement
NU	Neonatal unit
ZSTORCH	Group of congenital infections (zika, syphilis, toxoplasmosis, others [mainly hepatitis A, B, and C and human immunodeficiency virus], rubella, cytomegalovirus, and herpes)
CNS	Central nervous system
ABEP	Brazilian Association of Research Companies (in English)
VDRL	Venereal Disease Research Laboratory
CSM	Cramped synchronized movements
SPSS	Statistical Package for the Social Sciences
HIV	Human immunodeficiency virus
