# Correction: Silva et al. Abnormal Neurologic and Motor Function in Newborns Treated for Congenital Syphilis. *Infect. Dis. Rep.* 2025, *17*, 34

**DOI:** 10.3390/idr17040075

**Published:** 2025-06-30

**Authors:** Bruna Silva, Luciana Friedrich, Graziela Biazus, Renata Bueno, Carla Almeida

**Affiliations:** 1 Physical Education, Physioterapy and Dance School, Federal University of Rio Grande do Sul, Porto Alegre 90010-150, Brazil; brunavitoriar99@gmail.com (B.S.); renatabueno97@gmail.com (R.B.); carlaskilhan@gmail.com (C.A.); 2 Pediatrics Department, Federal University of Rio Grande do Sul, Porto Alegre 90010-150, Brazil; 3 Neonatology Service, Hospital de Clínicas of Porto Alegre, Porto Alegre 90035-903, Brazil; 4 Physioterapy Service, Hospital de Clínicas of Porto Alegre, Porto Alegre 90035-903, Brazil; gbiazus@hcpa.edu.br

In the original publication [1], there was an error regarding the affiliation for Luciana Friedrich. The original affiliation 2 should be split into two different affiliations: Pediatrics Department, Federal University of Rio Grande do Sul, Porto Alegre 90010-150, Brazil, and Neonatology Service, Hospital de Clínicas of Porto Alegre, Porto Alegre 90035-903, Brazil. The authors state that the scientific conclusions are unaffected. This correction was approved by the Academic Editor. The original publication has also been updated.
